# LncRNA-mir3471-*limd1* regulatory network plays critical roles in HIBD

**DOI:** 10.1007/s00221-023-06755-x

**Published:** 2023-12-26

**Authors:** Li Sun, Jun Wan, Bin Sun, Qiuyan Tian, Mei Li, Li-Xiao Xu, Chen-Xi Feng, Xiao Tong, Xing Feng, Xiaofeng Yang, Xin Ding

**Affiliations:** 1 Soochow Key Laboratory of Prevention and Treatment of Child Brain Injury;, Children’s Hospital of Soochow University, Suzhou, 215025 China; 2 Department of Pediatrics, Affiliated Hospital of Jiangnan University, Wuxi, China; 3 Department of Neonatology, Children’s Hospital of Soochow University, No.92 Zhongnanjie Road, Suzhou, 215025 Jiangsu China; 4 Pediatrics Research Institute, Children’s Hospital of Soochow University, Suzhou, 215025 China

**Keywords:** HIBD, lncRNA, miR-3471, limd1

## Abstract

The purpose of this study was to identify the target genes of tcon_00044595, elucidate its activation site, and provide novel insights into the pathogenesis and treatment of neonatal hypoxic–ischemic brain damage (HIBD). Through homologous blast analysis, we identified predicted target sequences in the neighboring regions of the long non-coding RNA (lncRNA) tcon_00044595, suggesting that limd1 is its target gene. Starbase was utilized to identify potential candidate microRNAs associated with the lncRNA. The interaction between the candidate microRNAs and limd1 was investigated and validated using various experimental methods including in vitro cell culture, cell transfection, dual fluorescence reporter detection system, and real-time PCR. Homology alignment analysis revealed that the lncRNA tcon_00044595 exhibited a 246 bp homologous sequence at the 3' end of the adjacent limd1 gene, with a conservation rate of 68%. Analysis conducted on Starbase online identified three potential microRNA candidates: miR-3471, miR-883a-5p, and miR-214-3p. Intracellular expression of the limd1 gene was significantly down-regulated upon transfection with miR-3471, while the other two microRNAs did not produce noticeable effects. Luciferase reporter assays identified two interaction sites (UTR-1, UTR-2) between miR-3471 and the limd1 3ʹUTR, with UTR-1 exhibiting a strong influence. Further CCK8 assay indicated a protective role of miR-3471 during low oxygen stroke in HIBD. The potential regulatory relationship between lncRNA (tcon_00044595), miR-3471, and the target gene limd1 suggests their involvement in the occurrence and development of HIBD, providing new insights for investigating the underlying mechanisms and exploring targeted therapeutic approaches for HIBD.

## Introduction

Neonatal hypoxic–ischemic brain damage (HIBD) refers to acute brain damage caused by perinatal hypoxia, which can result in long-term neurological consequences such as cerebral palsy and cognitive impairments, particularly in severe cases (Xiao et al. 2023; Chen et al. 2023). However, clinical manifestations often go unnoticed in milder cases. Several clinical studies have reported abnormal sleep characteristics in most children with HIBD (Pu et al. 2021; Tian et al. 2021). Our previous research indicated a close association between the presence of pineal cysts and sleep problems, as well as circadian rhythm dysfunction, in children with mild to moderate HIBD (Ding et al. 2016). The pineal gland, responsible for regulating circadian rhythms in vertebrates (Chauhan et al. 2023), may play a significant role in circadian rhythm disorders following neonatal HIBD, although the exact mechanism remains unknown.

Non-coding RNAs (ncRNAs) constitute the majority of the human transcribed genome. Increasing evidence suggests that post-transcriptional mechanisms, including ncRNAs, are crucial in regulating the expression of circadian genes within the pineal gland (Zhou et al. 2019a, b; Fu et al. 2022). Notably, miR-182 and miR-483, abundantly expressed in the pineal gland, target key regulators of the biological clock, namely Clock and Aanat (a rate-limiting enzyme involved in melatonin synthesis), respectively (Ding et al. 2015; Clokie et al. 2012). In a rat model of HIBD, up-regulation of miR-325 suppresses Aanat and melatonin production, disrupting circadian rhythms in children with HIBD (Yang et al. 2017). Sha Ning et al. further identified LHX3 as a downstream target of miR-325, where miR-325 knockout mice exhibited an miR-325-dependent physiological expression pattern of the transcription factor LHX3 in the pineal gland (Sha et al. 2021). In our previous study, we identified a highly enriched long non-coding RNA (lncRNA: tcon_00044595) in the pineal gland, demonstrating a circadian expression pattern. In vivo suppression of lncRNA: tcon_00044595 up-regulation significantly reduced the overactivation of pineal gland clock genes following HIBD, while in vitro inhibition of lncRNA: tcon_00044595 in cultured pineal cells, combined with miR-182, effectively mitigated clock overactivation after oxygen–glucose deprivation (OGD) (Li et al. 2020). These findings shed light on novel pathophysiological mechanisms underlying pineal gland dysfunction following neonatal HIBD. However, the downstream molecular mechanism remains elusive.

Building upon previous studies, in this study, we conducted homology comparisons, TargetScan software predictions, Starbase online analysis, and in vitro functional validation to identify downstream target genes of lncRNA: tcon_00044595, aiming to provide novel insights and directions for exploring the pathogenesis of circadian rhythm abnormalities in HIBD and potential gene-targeted therapies.

## Materials and methods

### Cells and reagents

The following materials were used in the study: DMEM/F12 medium (Procell, pm150210), DMEM high glucose medium (Procell, 150,310), fetal bovine serum (Gibco, 10,099,141), trypsin, streptomycin, and penicillin (Gibco). The miRNA qRT-PCR Starter Kit (TakaRa), forward primer (TakaRa), RT primer (TakaRa), and miRNA mimics (genephma) were used. Lipofectamine™ 3000 (Invitrogen) was employed for transfection. The dual luciferase reporter gene kit and reporter gene vector were obtained from Promega.

### RNA isolation, reverse transcription, and real-time PCR

The cells were isolated and rapidly frozen in liquid nitrogen. Total RNA was extracted using Trizol reagent (Life Technologies). For the microRNA expression assay, the miRNeasy Tissue/Cells Advanced Kit (Qiagen, 217,684) was used to extract total microRNAs. The TaqMan MicroRNA Assay Kit (Thermo Fisher) was employed to detect the expression levels of miR-3471.

TargetScan software predictions revealed two target sites for miR-3471 in the 3' untranslated region (UTR) of Limd1. For the luciferase reporter gene analysis, we constructed plasmids containing either a full-length 3ʹUTR fragment of Limd1 or a plasmid with an 8-bp deletion in the same region. Subsequently, HEK293T cells were transfected using the pMIreporter-luc VECTOR (TAKARA). Luciferase activity was assessed using the Dual-Glo® Luciferase Assay System (PROMEGA), and the normalized activity levels were compared to those of the pMIreporter-luc empty vector control.

### CCK8 assay

Neuroblastoma cell line PC12 was obtained from Shanghai National Cell Bank and maintained in DMEM medium supplemented with 10% horse serum (Gibco). Cells were cultured in a humidified incubator at 37 °C and 5% CO2.

After cell abundance reached 80%, the original medium was sucked out and cleaned with 1 × PBS twice. Then, they were replaced with EBSS and placed in the cell anoxic chamber (O2 concentration 1%, CO2 concentration 5%, N2 concentration 95%). After 2 h, the medium was removed and immediately replaced with the original medium. The incubation time for the treatment was 48 h. At the end of the treatment period, the culture medium was carefully aspirated from each well. Cells were washed once with PBS to remove any residual compounds.CCK-8 solution was prepared by diluting the CCK-8 reagent in fresh DMEM medium. The prepared CCK-8 solution was added to each well, ensuring complete coverage of the cells. Following the incubation period, the absorbance of the formazan dye was measured using a microplate reader. The absorbance values obtained from each well were recorded. Background absorbance was subtracted from the absorbance of each sample well. The relative cell viability or proliferation was calculated by normalizing the absorbance values to the control group. Data were expressed as mean ± SEM of three independent experiments.

## Results

### Target gene prediction of lncRNA tcon_00044595

**Fig. 1:**
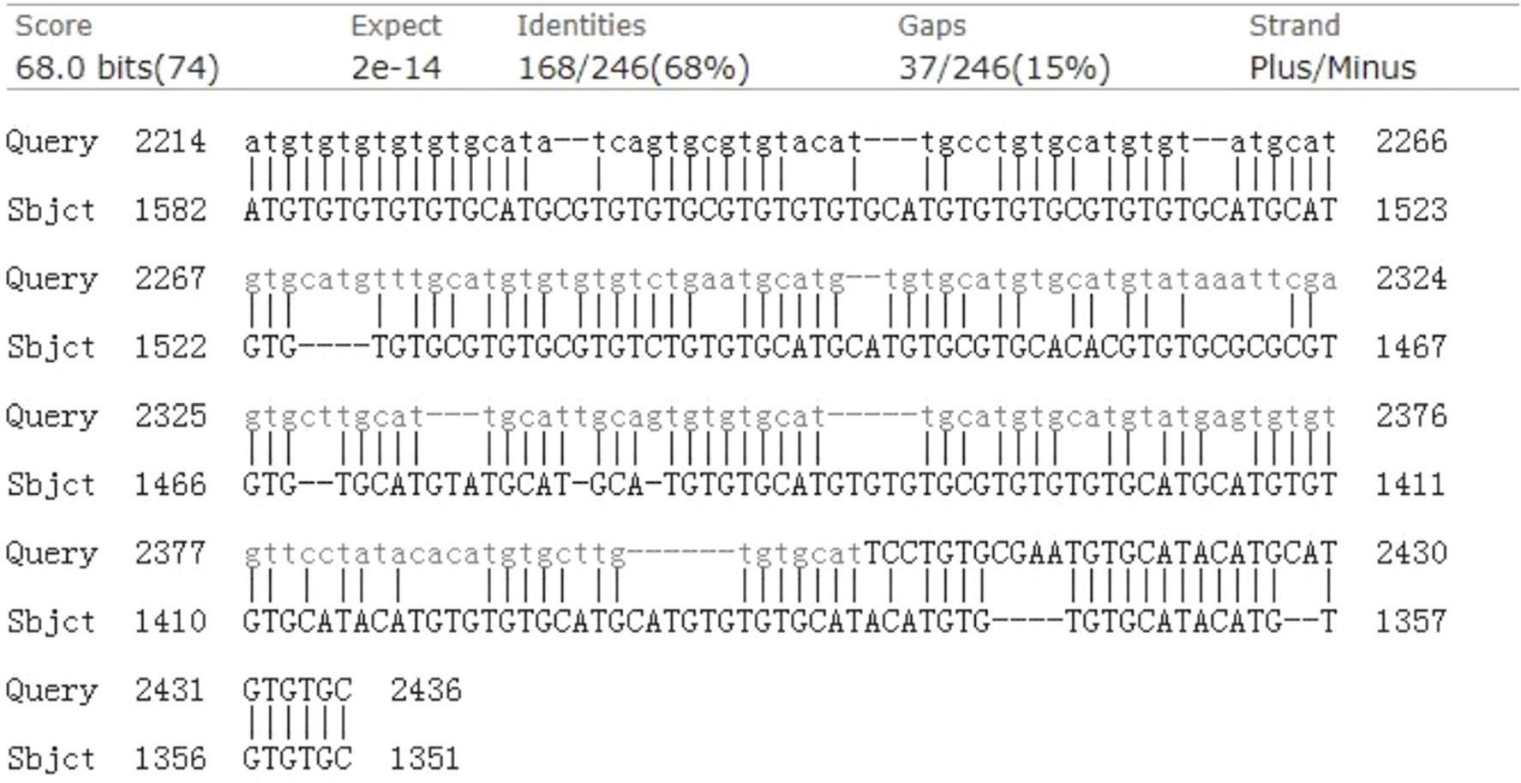
Identification of the target gene of lnc595 by homologous searching

In our previous study, we identified a highly enriched lncRNA: tcon_00044595 in the pineal gland of HIBD patients, which displayed a circadian expression pattern. To explore potential downstream target genes, we discovered a 246 bp homologous sequence between lncRNA: tcon_00044595 and the neighboring region of the limd1 gene within the 3ʹ-UTR. This homologous sequence exhibited a conservation rate of 68%, suggesting a potential targeting relationship between lncRNA: tcon_00044595 and limd1 (Fig. 1).

**Fig. 2:**
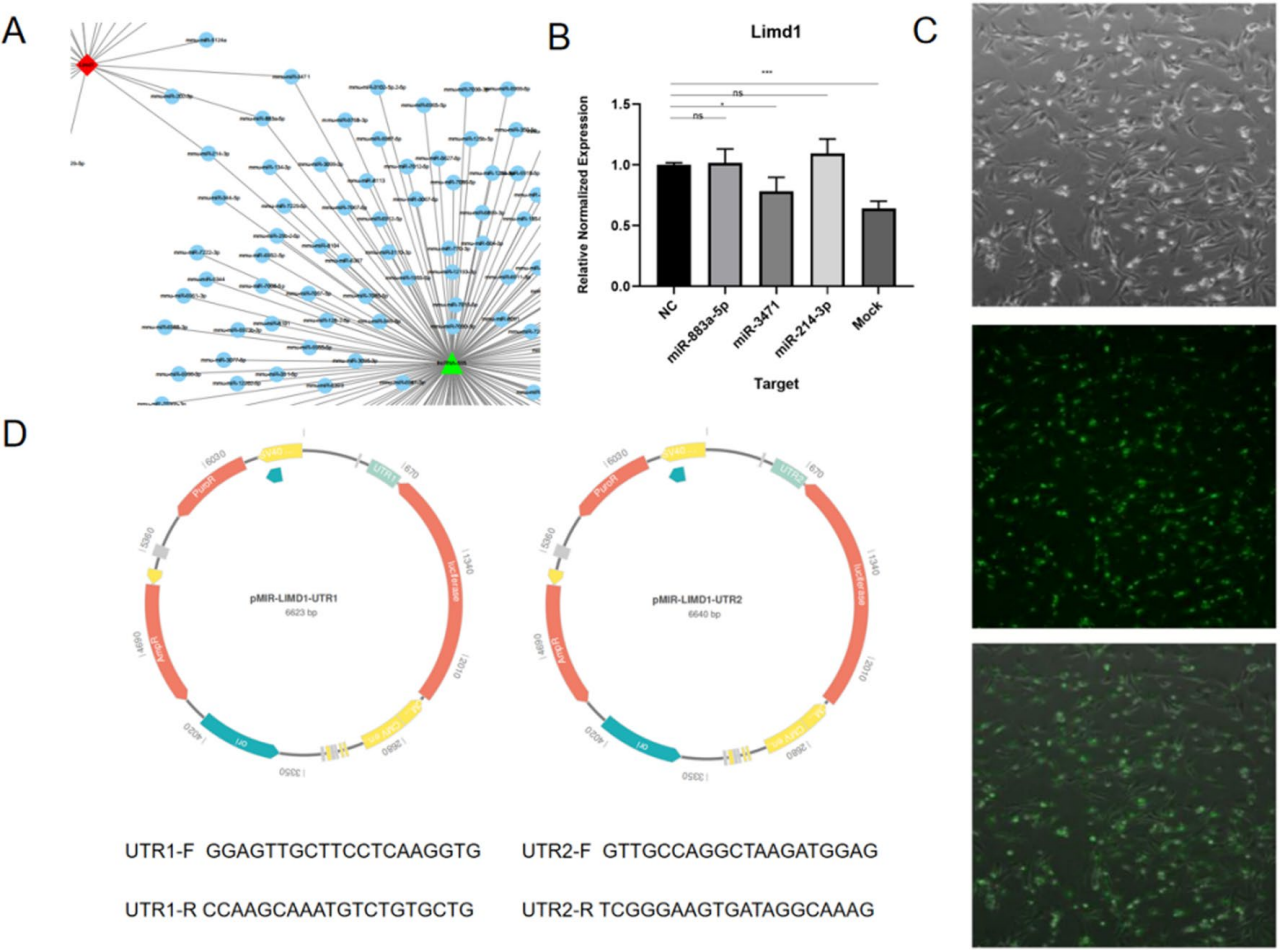
**A** Starbase online analysis to locate three candidate microRNAs. **B** Limd1 expression after transfection of three microRNAs, respectively (*n* = 3), a significant down-regulation of the limd1 gene in cells transfected with miR-3471, *p* < 0.05,*. **C** Transfection efficiency of miRNA. **D** Carrier construction

To gain further insights into the functions of lncRNA: tcon_00044595, we utilized the Starbase online analysis prediction system, which identified three candidate microRNAs: miR-3471, miR-883a-5p, and miR-214-3p (Fig. 2a). To validate if these candidate miRNAs directly regulated the expression of limd1, we synthesized analogs of the three miRNAs and individually transfected them into 293T cells. After 48 h, we quantified the expression of limd1 using fluorescence quantitative PCR. The results demonstrated a significant down-regulation of the limd1 gene in cells transfected with miR-3471, while miR-883a-5p and miR-214-3p had no notable effect on limd1 gene expression (Fig. 2b). Consequently, we identified miR-3471 as the target gene for further functional testing.

### Characterization of miR-3471 interaction with Limd1 3ʹUTR and identification of a specific target site

**Fig. 3:**
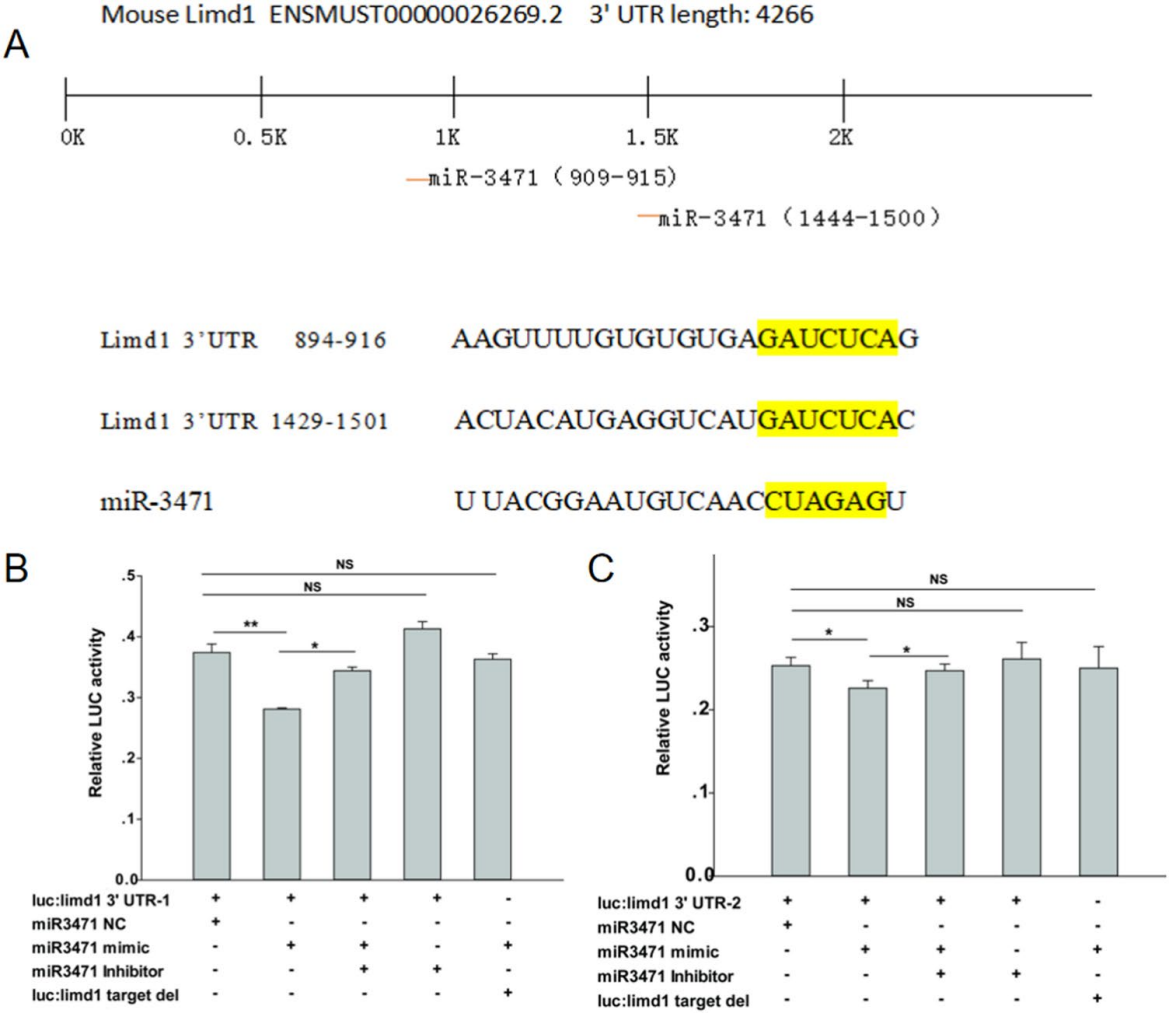
Identification of the target site of miR3471 in limd1 3ʹUTR. **A** Two candidate target sites of miR3471 in limd1 3ʹUTR were detected using TargetScan analysis. **B**, **C** Luciferase report assay shows the predicted site 1 was the real target site of miR3471

According to TargetScan predictions, the Limd1 3ʹUTR contains two target sites for miR-3471, located approximately 500 bp and 900 bp downstream. To investigate these interactions, we cloned the two target sites and inserted them into the pMIreportor vector, naming them UTR-1 and UTR-2, respectively. Subsequently, we transfected 293T cells to assess luciferase expression signals. The expression of both target sites decreased upon transfection with the miR-3471 mimic and increased when co-transfected with the inhibitor. Upon mutation of the DNA fragment containing the target sequence, the mimic showed no effect on luciferase expression. These findings suggest that miR-3471 interacts with a specific site within the Limd1 3ʹUTR. Notably, UTR-1 exhibited a stronger and statistically significant effect compared to UTR-2, indicating that miR-3471 has a specific interaction site with Limd1 at approximately 500 bp downstream (Fig. 3a, b).

### MiR3471 targets limd1 and plays a critical role in cellular activity under oxygen stroke

**Fig. 4:**
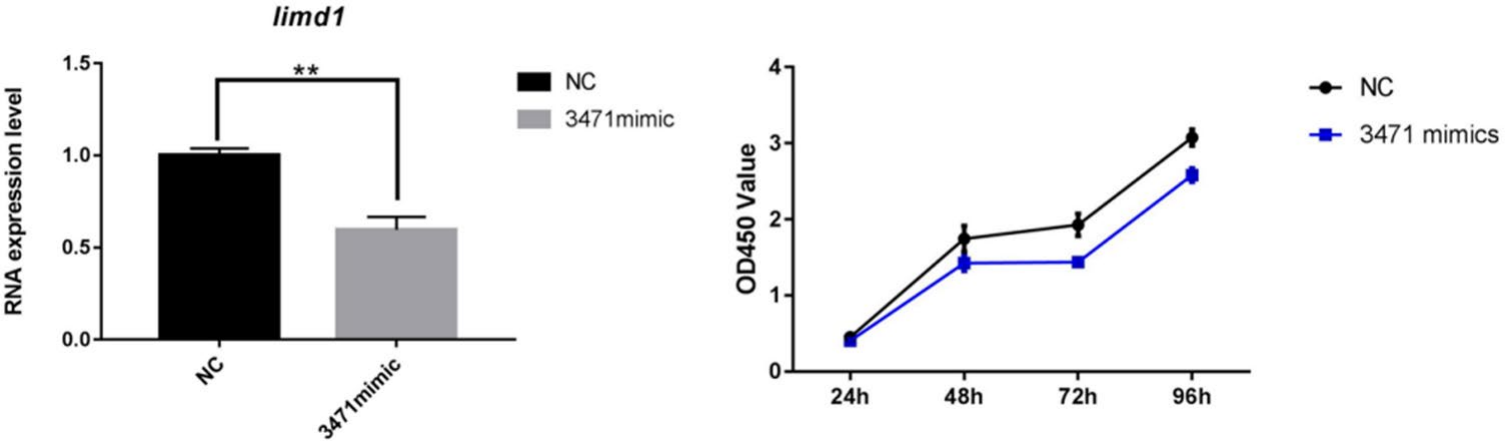
An overexpression of miR3471 by transfection of miR3471 mimics in PC12 cells results in a down-regulation of limd1 gene’s expression, *n* = 3, *p* < 0.05,**. **B** CCK8 analysis shows that the cell activities of PC12 are significantly decreased after oxygen stroke when transfected with miR3471 mimics

To further validate the impact of miR-3471 on limd1 in an in vivo setting, we employed the mouse neuroblastoma cell line PC12 and conducted transfection experiments using Lipofectamine 2000 to introduce miR-3471 mimics. This enabled us to examine the direct influence of miR-3471 on limd1. Subsequently, we collected the cells after a 48-h incubation period and extracted total RNA to determine the mRNA expression level of limd1 through qPCR analysis. The outcomes are presented in Fig. 4A, clearly indicating a significant reduction in the expression level of limd1 after transfection with miR-3471 mimics. These findings reinforce the role of miR-3471 in regulating limd1 and underscore its potential significance in related biological processes.

To elucidate the impact of miR3471 on cell viability in a simulated hypoxic–ischemic brain injury (HIBD) setting, we subjected PC12 cell lines to a 48-h hypoxic treatment. Subsequently, we transfected the cells with miR3471 mimics and control molecules and assessed cell activity using the CCK8 assay at 24, 48, 72, and 96 h post-treatment. The findings, depicted in Fig. 4B, revealed a consistent and significant decrease in cell activity for the miR3471-transfected cells compared to the control group across all time points. These results indicate that the expression of miR3471 plays a crucial role in the recovery of cell activity following HIBD, thereby highlighting its potential significance as a therapeutic target for mitigating the detrimental effects of HIBD.

## Discussion

Neonatal hypoxic–ischemic brain damage (HIBD) is a significant cause of morbidity and mortality in newborns, with long-term neurological deficits being a common outcome for surviving infants (Mohsenpour et al. 2021; Min et al. 2020; Greco et al. 2020). Despite advancements in medical imaging and clinical assessment, specific diagnostic indicators and targeted treatment options for HIBD are still lacking. Therefore, there is a pressing need to further understand the underlying mechanisms of HIBD development to improve diagnostic accuracy and explore potential therapeutic strategies.

MicroRNAs (miRNAs) have emerged as crucial regulators of gene expression at the post-transcriptional level and have been implicated in various physiological and pathological processes, including cerebral ischemia and hypoxia (Ponnusamy and Yip 2019; Shen and Ma 2020; Casey et al. 2020; Peeples 2023; Meng et al. 2021; Zhang et al. 2020). In this study, we investigated the role of miRNAs in HIBD, specifically focusing on miR-3471. Our results demonstrate that miR-3471 significantly down-regulated the expression of the Limd1 gene, suggesting a potential regulatory relationship between miR-3471 and Limd1 in HIBD.

Limd1, a LIM domain-containing protein, has been extensively studied in various cancers, where it has shown negative effects on tumor growth (Foxler et al. 2018; Zhou et al. 2019a, b; Huggins et al. 2017; Huggins and Andrulis 2008; Sharp et al. 2008; Liu et al. 2021). However, its involvement in HIBD has been largely unexplored. Our findings provide novel insights into the association between Limd1 and HIBD, indicating a potential role for Limd1 in the pathophysiology of HIBD. Moreover, we observed that Limd1 exhibits rhythmic expression patterns, raising the intriguing possibility that it may be involved in circadian rhythm disturbances following HIBD. Future studies should focus on elucidating the exact mechanisms underlying the involvement of Limd1 in HIBD-related circadian rhythm disruption.

Furthermore, we identified a putative interaction site between miR-3471 and the 3' untranslated region (3ʹUTR) of the Limd1 gene. Using luciferase reporter gene analysis, we confirmed that miR-3471 indeed interacts with this specific site within the Limd1 3ʹUTR. The functional significance of this interaction warrants further investigation, as it may shed light on the regulatory pathways involved in HIBD pathogenesis.

It is worth noting that our study also revealed the presence of other candidate miRNAs, including miR-883a-5p and miR-214-3p, which were predicted to potentially regulate Limd1. However, our experimental results indicated that only miR-3471 exhibited a significant down-regulatory effect on Limd1 expression. These findings suggest the specificity of miR-3471 in targeting Limd1 and highlight its potential as a therapeutic target for HIBD.

The identification of miR-3471 as a potential regulator of Limd1 and its interaction with specific sites in the Limd1 3ʹUTR expands our understanding of the molecular mechanisms underlying HIBD. However, several aspects of this regulatory axis remain to be explored. For instance, the functional consequences of miR-3471-mediated Limd1 down-regulation in the context of HIBD need to be investigated, as well as the downstream signaling pathways and biological processes influenced by this interaction. Additionally, it is essential to determine the broader regulatory network involving miR-3471 and its potential cross-talk with other miRNAs or factors in HIBD.

There are some limitations of this study. This is a preliminary study mainly based on bioinformatic analysis and in vitro experiments, findings from these experiments may not fully represent the complexities of in vivo conditions, and their clinical relevance might be limited. Besides, although the relationship between lncRNA (tcon_00044595), miR-3471, and limd1 in HIBD was discussed, the comprehensive mechanistic understanding of how these molecules are involved in the pathogenesis of HIBD is still unclear.

## Conclusions

Our study provides evidence for the involvement of miR-3471 and Limd1 in HIBD pathogenesis. The interaction between miR-3471 and the Limd1 gene, along with the presence of a rhythmic expression pattern for Limd1, opens new avenues for understanding the molecular mechanisms underlying HIBD and associated circadian rhythm disturbances. Further investigations are warranted to elucidate the functional consequences of miR-3471-mediated Limd1 regulation and to explore the potential therapeutic implications of targeting this regulatory axis in the management of HIBD.

## Data Availability

The data generated and analyzed for this current study are available from the corresponding author on reasonable request.
